# High burden of complicated skin and soft tissue infections in the Indigenous population of Central Australia due to dominant Panton Valentine leucocidin clones ST93-MRSA and CC121-MSSA

**DOI:** 10.1186/s12879-017-2460-3

**Published:** 2017-06-07

**Authors:** Susan A.J. Harch, Eleanor MacMorran, Steven Y.C. Tong, Deborah C. Holt, Judith Wilson, Eugene Athan, Saliya Hewagama

**Affiliations:** 1 Alice Springs Hospital, Alice Springs, Northern Territory Australia; 2 Menzies School of Health Research, Charles Darwin University, Darwin, Northern Territory Australia; 3 Deakin University, Geelong, VIC Australia; 4 SA Pathology, PO Box 14, Rundle Mall, Adelaide, South Australia 5000 Australia; 5 Royal Darwin Hospital, Darwin, Northern Territory Australia

**Keywords:** *Staphylococcus aureus*, Methicillin resistance, Abscess, Panton Valentine leucocidin

## Abstract

**Background:**

Superficial skin and soft tissue infections (SSTIs) are common among the Indigenous population of the desert regions of Central Australia. However, the overall burden of disease and molecular epidemiology of *Staphylococcus aureus* complicated SSTIs has yet to be described in this unique population.

**Methods:**

Alice Springs Hospital (ASH) admission data was interrogated to establish the population incidence of SSTIs. A prospective observational study was conducted on a subset of *S. aureus* complicated SSTIs (carbuncles and furuncles requiring surgical intervention) presenting during a one month period to further characterize the clinical and molecular epidemiology. High resolution melting analysis was used for clonal complex discrimination. Real-time polymerase chain reaction identifying the *lukF* component of the Panton Valentine leucocidin (*pvl)* gene determined *pvl* status. Clinical and outcome data was obtained from the ASH medical and Northern Territory shared electronic health records.

**Results:**

SSTIs represented 2.1% of ASH admissions during 2014. 82.6% occurred in Indigenous patients (*n* = 382) with an estimated incidence of 18.9 per 1, 000 people years compared to the non-Indigenous population of 2.9 per 1000, with an incident rate ratio of 6.6 (95% confidence interval 5.1–8.5). Clinical and molecular analysis was performed on 50 isolates from 47 patients. Community-associated methicillin-resistant *S. aureus* (CA-MRSA) predominated (57% of isolates). The high burden of SSTIs is partly explained by the prevalence of *pvl* positive strains of *S. aureus* (90% isolates) for both CA-MRSA and methicillin-susceptible *S. aureus* (MSSA). ST93-MRSA and CC121-MSSA were the most prevalent clones. SSTIs due to ST93-MRSA were more likely to require further debridement (*p* = 0.039), however they also more frequently received inactive antimicrobial therapy (*p* < 0.001).

**Conclusions:**

ST93-MRSA and CC121-MSSA are the dominant causes of carbuncles and furuncles in Central Australia. Both of these virulent clones harbor *pvl* but the impact on clinical outcomes remains uncertain. The high prevalence of CA-MRSA supports empiric vancomycin use in this population when antimicrobial therapy is indicated. Prompt surgical intervention remains the cornerstone of treatment.

## Background

Indigenous populations worldwide suffer from a high burden of infectious diseases. As with Indigenous populations elsewhere, Indigenous Australians of Central Australia share many of the same issues of remoteness, overcrowding, poverty and poor access to sanitation and health care [1–3].

Skin and soft tissue infections (SSTIs) are observed to be extremely common within the Indigenous population of Central Australia. The combination of socioeconomic disadvantage, high rates of communicable infections such as scabies and non-communicable diseases including diabetes mellitus provide a highly vulnerable host for SSTIs [2, 3]. *Staphylococcus aureus* infection is responsible for significant morbidity and mortality amongst this population, with SSTIs the leading cause of *S. aureus* bacteraemia [4] and frequently associated with prolonged hospital admissions for wound care with subsequent community dislocation. Despite this, the burden of disease due to superficial SSTIs in the Central Australian Indigenous population has not previously been investigated.

Community-associated methicillin-resistant *S. aureus* (CA-MRSA) and Panton Valentine leucocidin (PVL) positive infection are prevalent and seemingly on the increase in regional studies on *S. aureus* bacteraemia [4–7]. The rise of this *S. aureus* phenotype may be contributing to the burden of SSTIs related disease [8]. While the molecular epidemiology of *S. aureus* has been described in Indigenous Australians in tropical northern Australia [9], data on the circulating *S. aureus* clones associated with SSTIs is lacking for the geographically and ethnically distinct desert regions of Central Australia. Furthermore, the correlates between the molecular epidemiology and clinical characteristics including outcomes of complicated *S. aureus* SSTIs is unknown.

Additionally, the prevalence of a newly defined species, *S. argenteus* [10] has not been established. This is of interest as *S. argenteus*, formerly known as *S. aureus* CC75, is common in the Indigenous population of tropical northern Australia [11].

## Methods

Alice Springs Hospital (ASH) is the sole hospital providing tertiary health services for Central Australia, a population of approximately 60, 000 over a catchment area of 1.6 million square kilometers [12]. The catchment region is predominantly within the Northern Territory but also extends into Western Australia, South Australia and Queensland. Approximately 44% of the population identify as Indigenous but represent over 70% of ASH inpatients.

Data pertaining to the Diagnosis-Related Group (DRG) ‘cellulitis, boil, furuncle, carbuncle and abscess’ for the calendar year 2014 at ASH was queried to establish the incidence of hospital admissions due to SSTIs [13]. Australian Bureau of Statistics (ABS) population data [14] was utilised to calculate the regional incidence of SSTIs. The ABS estimate the total population for the Alice Springs, Barkly, Central Desert and MacDonnell Local Government Areas to be 48, 079 persons, with 20,262 persons identifying as Indigenous and 27,817 persons identifying as non-Indigenous [14].

We conducted a prospective, observational cohort study of a subset of patients presenting to ASH with a complicated, abscess related SSTI during a 1 month period (October 2014). We identified cases via operating theatre lists and included patients with: (1) spontaneous SSTI due to a carbuncle or furuncle, (2) surgical intervention required, and (3) available *S. aureus* isolate. Exclusion criteria were: (1) secondary infection related to a wound, and (2) polymicrobial infections.

We obtained microbiological cultures from routine clinical specimens, with preference for surgical specimens. *S. aureus* was identified by routine laboratory protocols (morphology, catalase and *Staphaurex* tests (Oxoid, 2011)), with organism confirmation and antimicrobial susceptibility testing performed on the *Vitek 2* (Biomerieux, version 7.01) using the Clinical Laboratory Standards Institute M100-S24 Performance Standards. We used real-time polymerase chain reaction (PCR) to detect the presence of the *nucA*, *mecA* and *lukSF* genes to determine the status of *S. aureus*, methicillin-resistance and PVL respectively. High resolution melting (HRM) analysis in conjunction with real-time PCR was used to discriminate the different clonal complexes [15]. We used ST93 rather than CC93 for those inferred to be ST93, as ST93 is a singleton sequence type with no identified related sequence types in the MLST database. We defined CA-MRSA as resistant to <3 non β–lactam antibiotic classes, and multi-resistant MRSA (mMRSA) as resistant to ≥3 non β–lactam antibiotic classes. Clinical data of cases was obtained from ASH medical records and included demographics, co-morbidities, recent hospitalization, infection, treatment and outcome data.

We analyzed differences between clonal types, clinical and outcome data using Analysis of Variance for continuous data and Fisher’s exact test for categorical variables in R version 3. 1. 2 (R Development Core Team 2014). Incident rate ratios were calculated in Stata version 14.2 (StataCorp, College Station, Texas).

Ethics approval was granted by the Central Australian Human Research Ethics Committee (HREC-14-223).

## Results

Skin and soft tissue infections represented by the DRG code ‘cellulitis, boil, furuncle, carbuncle and abscess’ constituted 2.1% of all ASH admissions during 2014. Consistent with previous years, it was the most frequent reason for admission under the General Surgical Unit. Overall, there were 462 ASH admissions attributed to SSTIs with the majority occurring in Indigenous patients (*n* = 382, 82.6%). The estimated incidence of SSTIs within the Indigenous population was 18.9 per 1, 000 people years and within the non-Indigenous population was 2.9 per 1, 000 people years, within an incident rate ratio of 6.6 (95% confidence interval 5.1–8.5).

Forty-nine cases for the prospective, observational cohort study were identified following screening and application of exclusion criteria. Most identified as Indigenous (90%), median age was 30 years and approximately 60% were female (Table 1). Despite the young population age, comorbidities with diabetes mellitus (50%), chronic kidney disease (25%), obesity (27%) and scabies infection (25%) were common. Almost half (49%) the population lived in remote communities.

**Table 1:** Correlation of clinical variables and *S. aureus* clonal complexes

Variable	Overall *n = 49* n (%^a^), median (IQR)	At least one isolate ST93 *n = 24* n (%), median (IQR)	At least one isolate CC121 *n = 15* n (%), median (IQR)	Other clonal complexes *n = 10* n (%), median (IQR)
Demographics				
Ethnicity: Indigenous	44 (89.8)	22 (91.7)	14 (93.3)	8 (80.0)
Gender: Female	29 (59.2)	12 (50.0)	9 (60.0)	8 (80.0)
Age (years)	30.0 [10.0, 46.0]	28.5 [11.2, 47.0]	34.0 [24.5, 45.5]	20.5 [4.5, 42.2]
Residence				
Town camp	12 (24.5)	6 (25.0)	4 (26.7)	2 (20.0)
Remote community	24 (49.0)	12 (50.0)	6 (40.0)	6 (60.0)
Prison	1 (2.0)		1 (6.7)	
Visitor	2 (4.1)	1 (4.2)		1 (10.0)
House in Alice Springs	10 (20.4)	5 (20.8)	4 (26.7)	1 (10.0)
Co-morbidities				
Diabetes mellitus	22 (44.9)	12 (50.0)	6 (40.0)	4 (40.0)
Chronic kidney disease	12 (24.5)	6 (25.0)	3 (20.0)	3 (30.0)
Haemodialysis	2 (4.1)	2 (8.3)	0 (0.0)	0 (0.0)
Excess alcohol use	6 (12.2)	3 (12.5)	2 (13.3)	1 (10.0)
Obesity (BMI ≥ 30)	13 (26.5)	3 (12.5)	7 (46.7)	3 (30.0)
Cardiac failure	6 (12.2)	2 (8.3)	3 (20.0)	1 (10.0)
Ischaemic heart disease	4 (8.2)	2 (8.3)	1 (6.7)	1 (10.0)
Scabies	12 (24.5)	5 (20.8)	4 (26.7)	3 (30.0)
Asthma	5 (10.2)	1 (4.2)	1 (6.7)	3 (30.0)
Healthcare exposure				
Admission in previous 6 months	18 (38.3)	9 (39.1)	5 (33.3)	4 (44.4)
Previous CA- MRSA	18 (38.3)	10 (43.5)	3 (20.0)	5 (55.6)
Clinical presentation				
Size (cm)	4.0 [3.0, 5.0]	4.0 [2.5, 5.0]	4.0 [3.0, 6.0]	5.5 [3.5, 9.0]
Duration (days)	5.0 [2.0, 7.0]	5.5 [3.2, 7.0]	4.0 [2.0, 6.5]	3.0 [2.0, 7.0]
Site of infection				
Head & neck	8 (16.3)	5 (20.8)	3 (20.0)	
Upper limb	9 (18.4)	7 (29.2)	1 (6.7)	1 (10.0)
Torso	6 (12.2)	3 (12.5)	13.3 (2)	
Buttock/groin/thigh	12 (24.5)	6 (25.0)	2 (13.3)	1 (10.0)
Lower leg	11 (22.4)	2 (8.3)	26.7 (4)	3 (30.0)
Multiple	3 (6.1)	1 (4.2)	13.3 (2)	5 (50.0)
Treatment				
Time to surgery (days)	1.0 [1.0, 1.0]	1.0 [1.0, 1.0]	1.0 [1.0, 1.0]	1.0 [1.0, 1.0]
Antimicrobials prior surgery				
Inactive	18 (36.7)	13 (54.2)		5 (50.0)
Active	30 (61.2)	10 (41.7)	15 (100.0)	5 (50.0)
Planned oral antibiotic duration (days)	7.0 [2.0, 7.0]	6.5 [3.2, 7.0]	5.0 [0.0, 7.0]	7.0 [5.5, 7.0]
Clinical outcomes				
Length of stay (days)	3.0 [2.0, 7.0]	3.0 [3.0, 6.0]	2.0 [2.0, 6.5]	3.0 [2.2, 7.0]
Progression to deep structures	5 (11.9)	3 (15.0)	0 (0.0)	2 (25.0)
Secondary infection	1 (2.5)	0 (0.0)	1 (7.7)	0 (0.0)
Return to theatre	6 (12.5)	6 (26.1)	0 (0.0)	0 (0.0)
Readmission within 30 days	4 (8.3)	2 (8.7)	2 (13.3)	0 (0.0)
Unscheduled medical review	8 (21.1)	4 (23.5)	2 (15.4)	2 (25.0)
Repeat antibiotics required	10 (26.3)	3 (17.6)	6 (46.2)	1 (12.5)
Antimicrobial susceptibility				
Methicillin resistance	28 (57.1)	23 (95.8)	0 (0.0)	5 (50.0)
Clindamycin resistance		3 (12.5)	9 (60.0)	
Trimethoprim/sulfamethoxazole resistance		0 (0.0)	0 (0.0)	

^a^Calculation of percentages takes into account missing data points

All cases had community onset disease, 36.7% had been admitted to ASH in the preceding 6 months and 38% had CA-MRSA previously isolated (clinical or screening specimens). CA-MRSA was the predominant phenotype (57%). Many patients received inactive empirical antimicrobial therapy (36.7%) prior to surgical intervention. The most frequently prescribed empiric antimicrobials were flucloxacillin (50%), vancomycin (20%) and cephazolin (14%). Surgical intervention was generally performed within 1 day of admission. Blood cultures were obtained in 16 cases (33%) and were positive for MSSA in just one instance.

Clonal complex and *pvl* status was successfully determined for 50 isolates from 47 patients. Eight clonal complexes were identified (Table 2). The clonal complexes with the highest frequency were ST93 (48%) and CC121 (30%). Of the paired isolates, one case had two MSSA strain types (CC5 and CC121) and two cases had concurrent MSSA and CA-MRSA strain types (CC5 and CC8, and CC1 and ST93). *pvl* positive disease dominated the cohort with 90% of isolates carrying the *lukSF* gene, occurring in both MSSA and CA-MRSA isolates and across all clonal complexes except for CC1 and CC88. *S. argenteus* was not identified.

**Table 2:** *S. aureus* isolates: Frequency of clonal complexes and carriage of *pvl* and mecA genes

	Clonal complex n (%)	*pvl* positive n (%)	mecA positive n (%)
Overall (*n* = 50)		45 (90.0)	28 (56.0)
CC1	1 (2.0)	0 (0)	0 (0)
CC5	4 (8.0)	3 (75.0)	3 (75.0)
CC8	3 (6.0)	1 (33.3)	1 (33.3)
CC22	1 (2.0)	1 (100.0)	0 (0)
CC30	1 (2.0)	1 (100.0)	1 (100.0)
ST93	24 (48.0)	24 (100.0)	23 (95.8)
CC121	15 (30. 0)	15 (100.0)	0 (0)
CC88	1 (2.0)	0 (0)	0 (0)

There was clear correlation between clones and antimicrobial phenotype. The majority of ST93 isolates were CA-MRSA (96%), whilst all CC121 isolates were MSSA (*p* < 0.001). 60% of CC121 isolates were resistant to clindamycin, compared to 12.5% of ST93 isolates being clindamycin resistant (*p* = 0.006). Trimethoprim/ sulfamethoxazole resistance occurred in two *pvl* positive CC5 isolates (4%).

There were some differences between *S. aureus* clones and clinical variables (Table 1). On univariate analysis, infections due to ST93 were more likely to require further debridement (*p* = 0.039) but this group also more frequently received inactive antimicrobial therapy prior to debridement (*p* < 0.001).

## Discussion

Key findings of this study include the very large burden of SSTIs experienced in Central Australia and the vast disparity between Indigenous and non-Indigenous Australians. The incidence of SSTIs requiring hospitalisation within the Indigenous population of Central Australia is estimated to be 18.8 per 1000 people years. This is almost seven times the incidence for the local non-Indigenous population. More concerning, the incidence is over 30 times the estimated national Australian hospitalisation rate for cutaneous abscesses of 62 per 100, 000 people years [16]. Moreover the incidence is significantly higher than other developed nations, with an American retrospective survey describing an inpatient admission SSTI incidence of 2.19 per 1, 000 person years [8].

There continues to be a rise in CA-MRSA particularly within Australian Indigenous communities [7] and our study reflects this trend. We have demonstrated that CA-MRSA is now the dominant *S. aureus* phenotype in carbuncle and furuncle related SSTIs in the desert regions of Central Australia. The prevalence of CA-MRSA in this SSTI subset (57%) well exceeds the prevalence rates of approximately 20% described in previous Australian *S. aureus* studies [6, 7, 17]. CA-MRSA is increasingly responsible for SSTIs in Australian Indigenous populations, and in particular the dominant ST93-MRSA clone has continued its spread within Australia into the desert regions of Central Australia. MSSA continues to contribute substantially to the burden of carbuncle and furuncle related SSTIs with CC121 the major MSSA clone. Notably, both ST93 and CC121 are *pvl* positive clones.

Despite the geographic isolation, high Indigenous population and substantial burden of disease, the molecular epidemiology of SSTI isolates in Central Australia had a number of similarities to other Australian regions. Clonal diversity is a unique feature of *S. aureus* in Australia, with a recent prevalence survey identifying more than 30 clones in CA-MRSA alone [17]. There are six major MRSA clones including ST93-IV, ST30-IV, ST1-IV, ST45-V, ST78-IV and ST5-IV [17]. ST93 is notable for causing abundant [9, 17, 18] and possibly more virulent disease in Australia [19] . We also observed significant clonal diversity with 8 clonal complexes identified within 50 isolates, including CC1, CC5, CC8, CC22, CC30, ST93, CC121 and CC88.

Like other regions in Australia, we observed ST93 to be the most frequent strain (48%) and strongly associated with CA-MRSA (96%). ST93 has been found to hyperexpress the exotoxin α-haemolysin which enhances the virulence of the clone, particularly in SSTIs [20]. The high virulence and expression of α-haemolysin in ST93 may be a driving factor in the substantial burden of disease seen in Central Australia [19–21].

Following ST93, the next major clone was CC121 (30%), which was exclusively methicillin-susceptible. The association of CC121 with SSTI is consistent with the international literature and CC121 is phenotypically usually MSSA [22]. The molecular epidemiology of MSSA infections has been studied in less detail than CA-MRSA [23], but CC121 is commonly associated with SSTIs in Australia. However, the high prevalence of CC121 in our study (30%) is greater than that reported elsewhere in the Northern Territory ‘Top End’ and metropolitan centers [9, 18], and may be a unique feature of the Central Australian region.

The absence of *S. argenteus* is an interesting finding of this study and key difference to other Australian regions. *S. argenteus* was initially reported in the Northern Territory ‘Top End’ [9, 24], but it is now clear it has a global distribution [10]. The clinical niche of *S. argenteus* appears to differ from *S. aureus* [21]. *S. argenteus* may have less of a predilection to cause abscesses and furuncles and further studies of non-complicated SSTI will be required to determine whether *S. argenteus* is truly absent from Central Australia.

The dominance of *pvl* positive *S. aureus* isolates (90%) may be contributing to the very high incidence of abscess related SSTIs in Central Australia [9] and is notable for two reasons. Firstly, it was widespread in both MSSA and CA-MRSA isolates. The association between PVL and abscess related SSTIs is well described [25], however the prevalence of *pvl* in our cohort was substantially higher than in other Australian urban settings (MSSA 15%, CA-MRSA 42%) [18] and the Northern Territory Top End (MSSA 40%, CA-MRSA 54%). Secondly, the presence of *pvl* appears to have limited impact on clinical outcomes following surgical intervention. Controversy continues regarding the pathogenic role of PVL, with laboratory and animal model data suggesting it is not a major virulence factor [26, 27]. In our study, detailed clinical outcome comparisons were limited by the small number of *pvl* negative cases (10%). Despite this, most patients achieved good clinical outcomes with only one episode of bacteraemia and a minority experiencing progression to deep tissue structures (12%) and secondary infection (2.5%). It was not possible to determine if these complications were related to the presence of *pvl* or a delay in presentation to health-services or poor wound care upon discharge. Therefore, we believe the typical rapid response to surgical intervention observed in our study provides further support to the findings of Shallcross et al. that provided ‘appropriate surgical treatment and the correct antibiotics’ are received, PVL appears to have little impact on clinical outcomes [25].

Patients with CA-MRSA frequently received inactive empiric antimicrobial therapy with antibiotic prescription often in accordance with the Australian Therapeutic Guidelines recommendation of di/flucloxacillin for empirical therapy [28]. It follows then that infections due to ST93 were more likely to receive inactive antimicrobials compared to CC121 related infections (54.2% vs 100%, *p* < 0.001). More importantly, all cases requiring further debridement (*n* = 6) were ST93 (*p* = 0.039) and 50% of these received inactive empiric antimicrobials. The high rates of CA-MRSA and potential impact on clinical outcomes lead us now to recommend the empiric use of vancomycin for patients in Central Australia presenting with SSTIs requiring surgical management. Trimethoprim-sulfamethoxazole may be a preferred empiric oral agent given the high rates of observed clindamycin resistance (32%) and dosing ease.

Associations between clonal complexes and antibiograms were noteworthy. Clindamycin resistance occurred in 32% of isolates, substantially higher than previous studies [17]. There was also a significant difference in clindamycin resistance between the major strains CC121 (60%) and ST93 (12.5%), *p* = 0.006. The impact on antimicrobial prescribing is attenuated by CC121 isolates being exclusively MSSA but this highlights the evolution to a more broadly resistance antibiogram. The rise in clindamycin resistance may be driven by high use of macrolide antibiotics in Indigenous communities of Central Australia, typically indicated for *Chlamydia trachomatis* or chronic suppurative lung disease.

There are now reports of resistance to trimethoprim-sulfamethoxazole in CC5 strains from Indigenous communities in the neighboring jurisdiction of Western Australia [29]. We found two CC5 trimethoprim-sulfamethoxazole resistant MRSA strains that were also *pvl* positive. Therefore, ongoing surveillance for changes in resistance and clonal patterns of *S. aureus* are required to update prescribing recommendations.

This study is limited by its observational design with reliance on coding data to determine SSTI incidence rates and retrieval of clinical data from medical records. It was not possible to obtain ABS population data for the entire ASH catchment given it includes partial Local Government Areas of Western Australia, South Australia and Queensland. Consequently, the population incidence rate may be overestimated. Correlation of the estimated ASH catchment population of 60, 000 with 44% identifying as Indigenous determines an incidence of SSTIs within the Indigenous population as 14.5 per 1, 000 people years and within the non-Indigenous population as 2.4 per 1000 people years.

The small sample size of cases, microbiological isolates and patients with PVL negative disease is the major limitation of this study and prevented detailed molecular and clinical correlations. Similarly, our sampling occurred over 1 month and if seasonal variation was present, we would not have identified this. Review of the geographic distribution of clonal complexes was not possible due to use of broad residential categories and the highly mobile nature of the local Indigenous population. Given asymptomatic patients colonized with *pvl* positive *S. aureus* were not included, associations with *pvl* positivity and clinical disease are only epidemiological in nature and not causal. Other recognized *S. aureus* virulence factors such as α-haemolysin were not assessed.

## Conclusions

In conclusion, we observed extremely high rates of complicated SSTIs requiring hospitalization in Central Australia, with the Indigenous population disproportionately affected. Detailed review of the molecular epidemiology of carbuncle and furuncle related SSTIs revealed the dominance of two clones ST93-MRSA and CC121-MSSA. These two virulent clones both possess the *pvl* gene but the impact of PVL toxin on clinical outcomes remains uncertain. The prevalence of CA-MRSA continues to rise locally, with significant ramifications for antimicrobial prescribing. Empiric intravenous vancomycin or oral trimethoprim-sulfamethoxazole is recommended for this population when antimicrobial therapy is indicated. Prompt surgical intervention remains the cornerstone of treatment. Further studies correlating clonal complex type, asymptomatic *pvl* carriage as well as the presence of other virulence factors will help improve our understanding of the pathogenesis of SSTIs.

## Abbreviations

ABS: Australian Bureau of Statistics
ASH: Alice Springs Hospital
CA-MRSA: Community-associated methicillin-resistant *Staphylococcus aureus*
DRG: Diagnosis-related group
HRM: High resolution melting (analysis)
mMRSA: Multi-resistant methicillin-resistant *Staphylococcus aureus*
MSSA: Methicillin-susceptible *Staphylococcus aureus*
PCR: Polymerase chain reaction
*Pvl*: Panton Valentine leucocidin (gene)
PVL: Panton Valentine leucocidin (toxin)
SSTIs: Skin and soft tissue infections

## References

[1] Tong SYC, McDonald MI, Holt DC, Currie BJ (2008). Global implications of the emergence of community-associated methicillin-resistant *Staphylococcus aureus* in Indigenous populations. Clin Infect Dis.

[2] Tong SYC, van Hal S, Einsiedel LJ, Currie BJ, Turnidge J (2012). Impact of ethnicity and socioeconomic status on *Staphylococcus aureus* bacteraemia incidence and mortality: a heavy burden in Indigenous Australians. BioMedCentral Infec Dis.

[3] Einsiedel LJ, Fernandes L, Joseph S, Brown A, Woodman RJ (2013). Non-communicable diseases, infection and survival in a retrospective cohort of Indigenous and non-Indigenous adults in Central Australia. BMJ Open.

[4] Hewagama S, Spelman T, Einsiedel L. *Staphylococcus aureus* Bacteraemia at Alice Springs Hopsital, Central Australia, 2003–2006. Intern Med J. 2011:505–12. 10.1111/j.1445-5994.2011.02449.x 21309994

[5] Hewagama S, Spelman T, Woolley M, McLeod JE, Gordon D, Einsiedel LJ (2016). The Epidemiology of *Staphylococcal aureus and* Panton-Valentine Leucocidin (pvl) in Central Australia, 2006–2010. BioMedCentral Infect Dis.

[6] Stevens C, Ralph A, McLeod JE, McDonald MI (2006). Community-acquired methicillin-resistant *Staphylococcus aureus* in Central Australia. Commun Dis Intell.

[7] Tong SYC, Varrone L, Chatfield MD, Beaman M, Giffard PM (2012). Progressive increase in community-associated methicillin-resistant *Staphylococcus aureus* in Indigenous populations in northern Australia from 1993 to. Epidemiol Infect.

[8] Miller LG, Eisenberg D, Liu H, Chang C, Wang Y, Luthra R, et al. Incidence of skin and soft tissue infections in ambulatory and inpatient settings, 2005-2010. BioMedCentral Infect Dis. 2015;15(362):1–8. 10.1186/s12879-015-1071-0 PMC4546168 26293161

[9] Tong SYC, Lilliebridge RA, Bishop EJ, Cheng AC, Holt DC, McDonald MI, et al. Clinical correlates of Panton-Valentine Leukocidin (PVL), PVL isoforms, and clonal complex in the *Staphylococcus aureus* population of northern Australia. J Infect Dis. 2010;202(5):760–9. 10.1086/655396 20662623

[10] Tong SYC, Schaumburg F, Ellington MJ, Corander J, Pichon B, Leendertz F, et al. Novel staphylococcal species that form part of a Staphyloccus aureus-related complex: the non-pigmented Staphylococcus argenteus sp nov and the non-human primate-associated Staphylococcus schweitzeri sp nov. Int J Syst Evol Microbiol. 2015;65:15–22. 10.1099/ijs.0.062752-0 PMC4298100 25269845

[11] Tong SYC, Bishop EJ, Lilliebridge RA, Cheng AC, Spasova-Penkova Z, Holt DC, et al. Community- Associated Strains of Methicillin- Resistant *Staphylococcus aureus* and Methicillin- Susceptible *S.aureus* in Indigenous Northern Australia: Epidemiology and Outcomes. J Infect Dis. 2009;199:1461–70. 10.1086/598218 19392622

[12] Northern Territory Department of Health: Alice Springs Hospital: https://health.nt.gov.au/professionals/medical-officers/teaching-hospitals/alice-springs-hospital. Accessed 3 June 2017.

[13] Alice Springs Hospital: Diagnosis-Related Group J64B - Cellulitis- CSCC: *Alice Springs Hospital Separation Data.* 2015.

[14] Australian Bureau of Statistics: Local Government Areas - Alice Springs, Barkly, Central Desert, MacDonnell: *Data by Region.* Edited by Australian Bureau of Statistics. Canberra, Australia; 2015.

[15] Lilliebridge RA, Tong SYC, Giffard PM, Holt DC. The Utility of High-Resolution Melting Analysis of SNP Nucleated PCR Amplicons - An MLST Based *Staphylococcus aurues* Typing Scheme. PLoS One. 2011:6(6). 10.1371/journal.pone.0019749 PMC3120814 21731606

[16] Vaska VL, Nimmo GR, Jones M, Grimwood K, Paterson DL (1999). Increases in Australian cutaneous abscess hospitalisations. Eur J Clin Microbiol Infect Dis.

[17] Coombs GW, Daley DA, Pearson JC, Nimmo GR, Collignon PJ, McLaws M-L, et al. Community-onset *Staphylococcal aureus* surveillance program annual report, 2012. Commun Dis Intell. 2014;38(1):E59–69. 10.33321/cdi.2014.38.12 25409357

[18] Bennett CM, Coombs GW, Wood GM, Howden BP, Johnson LEA, White D, et al. Community-onset *Staphylococcal aureus* infections presenting to general practices in South-eastern Australia. Epidemiol Infect. 2014;2014(142):501–11. 10.1017/S0950268813001581 PMC9151107 23866772

[19] Chua K, Seemann T, Harrison P, Monagle S, Korman T, Johnson PDR, et al. The Dominant Australian Community-Acquired Methicillin-Resistant *Staphylococcus aureus* Clone ST93-IV[2B] Is Highly Virulent and Genetically Distinct. *PLOS One*. 2011;6(10):e25887. 10.1371/journal.pone.0025887 PMC3185049 21991381

[20] Chua K, Monk I, Lin Y-H, Seemann T, Tuck KL, Porter J, et al. Hyperexpression of α-haemolysin explains enhanced virulence of sequence type 93 community-associated methicillin-resistant *Staphylococcus aureus*. BioMedCentral Microbiol. 2014:**14**(31). 10.1186/1471-2180-14-31 PMC3922988 24512075

[21] Tong SYC, Sharma-Kuinkel B, Thaden JT, Whitney A, Yang S-J, Mishra NN, et al. Virulence of Endemic Nonpigmented Northern Australian *Staphylococcus aureus* Clone (Clonal Complex 75, *S.argenteus*) Is Not Augmented by Staphyloxanthin. J Infect Dis. 2013;2013(208):520–7. 10.1093/infdis/jit173 PMC3699000 23599317

[22] Monecke S, Coombs GW, Shore AC, Coleman DC, Akpaka P (2011). A field guide to pandemic, epidemic and sporadic clones of methicillin-resistant *Staphylococcus aureus*. PLoS One.

[23] Williamson DA, Coombs GW, Nimmo GR (2014). *Staphylococcus aureus* 'Down Under': contemporary epidemiology of *S. aureus* in Australia, New Zealand, and the South West Pacific. Clin Microbiol Infect.

[24] Ng J, Holt DC, Lilliebridge RA, Stephens AJ, Huygens F, Tong SYC, et al. Phylogenetically distinct *Staphylococcus aureus* lineage prevalent among Indigenous communities in northern Australia. J Clin Microbiol. 2009;47(7):2295–300. 10.1128/JCM.00122-09 PMC2708510 19420161

[25] Shallcross L, Fragazy E, Johnson AM, Hayward AC (2013). The role of Panton-Valentine leucocidin toxin in staphylococcal disease: a systematic review and meta-analysis. Lancet Infect Dis.

[26] Tong A, Tong SYC, Zhang Y, Lamlertthon S, Sharma-Kuinkel B, Rude T, et al. Panton-Valentine Leukocidin is not the primary determinant of outcome for *Staphylococcus aureus* skin infections: evaluation from the CANVAS studies. PLoS One. 2012;7(5):e37212. 10.1371/journal.pone.0037212 PMC3356380 22623995

[27] Hay R, Noor NM (2011). Panton-Valentine leucocidin and severe *Staphylococcus aureus* infections of the skin: sole culprit or does it have accomplices. Curr Opin Infect Dis.

[28] Moulds R, Anderson T, Daly C, Daskalakis S, Evill C, Grayson L, Korman T: Boils and carbuncles. In: *Therapeutic Guidelines: Antibiotics.* Edited by Moulds R, etg45 Therapeutic Guidelines; 2014.

[29] Coombs GW, Pearson J, Robinson O. Western Australian Methicillin-Resistant Staphylococcus aureus (MRSA) Epidemiology and Typing Report. July 1 2013 to June 30 2014. In: Gram-positive bacteria Typing laboratory, PathWest laboratory medicine, and Australian collaborating Centre for Enterococcus and Staphylococcus Species (ACCESS) Typing and Research, Murdoch and Curtain Universities, Perth 2015.

